# Plasma total cell-free DNA is a prognostic biomarker of overall survival in metastatic solid tumour patients

**DOI:** 10.1038/s41416-019-0491-9

**Published:** 2019-06-12

**Authors:** Ida Viller Tuxen, Lise Barlebo Ahlborn, Morten Mau-Soerensen, Kristoffer Staal Rohrberg, Finn Cilius Nielsen, Olga Oestrup, Christina Westmose Yde, Ivan Richter Vogelius, Ulrik Lassen

**Affiliations:** 1 The Phase I Unit, Department of Oncology, Copenhagen University, Rigshospitalet, Copenhagen, Denmark; 2 Center for Genomic Medicine, Copenhagen University, Rigshospitalet, Copenhagen, Denmark; 3 Section of Radiotherapy, Department of Oncology, Copenhagen University, Rigshospitalet, Copenhagen, Denmark

**Keywords:** Cancer genomics, Molecular biology

## Abstract

**Background:**

Selecting patients for early clinical trials is a challenging process and clinicians lack sufficient tools to predict overall survival (OS). Circulating cell-free DNA (cfDNA) has recently been shown to be a promising prognostic biomarker. The aim of this study was to investigate whether baseline cfDNA measurement could improve the prognostic information of the Royal Marsden Hospital (RMH) score.

**Methods:**

Solid tumour patients referred for phase I trials were included in the Copenhagen Personalized Oncology (CoPPO) programme. Baseline characteristics were collected prospectively, including the RMH prognostic score, Eastern Cooperative Oncology Group (ECOG) performance status and concentration of cfDNA per millilitre plasma. Cox proportional hazards model was used to assess the prognostic value of baseline variables.

**Results:**

Plasma cfDNA concentration was quantifiable in 302 patients out of a total of 419 included in the study period of 2 years and 5 months. The RMH score was confirmed to be associated with OS. Cell-free DNA was shown to be an independent prognostic marker of OS and improved the risk model, including RMH, performance status and age. Furthermore, both plasma cfDNA concentration and RMH score were associated with treatment allocation (*p* < 0.00001).

**Conclusion:**

Our model based on RMH score, age, ECOG performance status and cfDNA improved prediction of OS and constitutes a clinically valuable tool when selecting patients for early clinical trials. An interactive version of the prognostic model is published on http://bit.ly/phase1survival.

## Background

Cancer patients with exhausted treatment options can be referred to phase 1 clinical trials where the primary objectives are dose finding and toxicity assessment. However, only a minority of patients will benefit from treatment^1,2^ and selection of the most appropriate patient for therapy is challenging. Generally used eligibility criteria include adequate organ function, good performance status and life expectancy more than 3 months. Clinicians often misjudge the survival of the patients leading to screen failures and waste of important time for the individual patient.^3–5^

Different prognostic scores have been introduced in order to select patients for phase 1 trials.^6,7^ The Royal Marsden Hospital (RMH) score has been validated in various phase 1 cohorts^8–11^ and consist of three variables: Elevated lactate dehydrogenase (LDH) (> upper limit), low serum-albumin (< 35 g/L) and more than two metastatic sites. Although, these prognostic scores have been validated, the clinical use is limited.

In recent years, much attention in oncological studies has been given to circulating cell-free DNA (cfDNA) as a non-invasive tumour marker used in diagnostics and treatment monitoring. Cell-free DNA is short fragments of DNA present in plasma and other body fluids and originates from apoptotic and necrotic cells representing normal tissue and potentially multiple tumour lesions.^12^ cfDNA has been highlighted as a new potential biomarker for overall survival (OS) in various cancer subtypes.^13–15^ To investigate the clinical utility of cfDNA in patients referred to phase 1 trials, we conducted a pre-planned examination of baseline plasma cfDNA levels in patients participating in the Copenhagen Prospective Personalized Oncology (CoPPO) trial.^16^ The aim of the study was to investigate whether additional measurements of plasma cfDNA concentration improved the prognostic value of the RMH score and thus selection of patients for phase I trials.

## Methods

### Patients and study design

This study included patients enrolled in the CoPPO study (NCT02290522) from October 2014–February 2017. The CoPPO study aims to investigate the clinical utility of molecular profiling to select patients for early clinical trials. All patients fulfilled the inclusion criteria including: exhausted treatment options, life expectancy ≥ 3 months, normal organ function, age ≥ 18 years, Eastern Cooperative Oncology Group (ECOG) performance status 0 or 1 and lesions assessable for biopsy. Basic characteristics including serum albumin and lactate dehydrogenase (LDH) were registered at inclusion. Values obtained within 2 months of inclusion were accepted.

The study was conducted in accordance with the Declaration of Helsinki and approved by the Danish Data Protection Agency and the Regional Ethics Committee (Danish Ethical Committee, file number: 1300530). All patients provided signed informed consent. Cut-off date was 22 February 2018. Examination of plasma cfDNA concentration was planned but the statistical analysis was not pre-specified and thereby considered as exploratory.

### Extraction and quantification of cfDNA

Peripheral blood was collected in cell-stabilising Blood Collection Tubes (BCT; Streck Laboratories, Omaha, NE, USA) and cfDNA was extracted from 2–4 millilitre (ml) plasma using the QIAsymphony Circulating DNA Kit (Qiagen, Hilden, Germany) according to the manufacturer’s instructions using an elution volume of 60 µl as previously described.^17^ Quantification of cfDNA was performed using the dsDNA HS Assay Kit (> 10 pg/µL) on a Qubit Fluorometer (Thermo Fisher Scientific, Waltham, MA) and the concentration of cfDNA per ml of plasma was calculated for each sample.^18^

For validation of the cfDNA quantification method, we measured total cfDNA in triplicates using the Agilent 4200 TapeStation system (D5000) for 49 samples with low cfDNA concentration (0–15 ng/ml plasma (A)) and 47 samples with high concentration (50–1000 ng/ml plasma (B)) (Fig. S2). For the high-concentration group we diluted samples (1:10) if the concentration was >100 ng/ml plasma. The Agilent 4200 TapeStation system (D5000) uses electrophoresis to separate DNA fragments from 100–5000 bp and can quantify DNA concentrations down to 0.1 ng/µL. In contrast, the Qubit system uses intercalating fluorescent dyes binding only double stranded DNA, leading to concentration measures around half the value of the ones from the TapeStation. This was supported by the Bland–Altman plot shown in Fig. S3 illustrating the concordance between Qubit and TapeStation cfDNA quantification (mean percentage difference between the methods of 21.8% and limits of agreement around ± 40%).

### Statistical analysis

OS (Kaplan–Meier) was calculated from the date of inclusion to time of death or censure. The prognostic value of the RMH score was tested using a univariate Cox model with RMH score as scale input. Subsequently, multivariable analysis was performed with performance status and RMH score as predefined categorical covariables. Age and the log-transformed value of cfDNA (logcfDNA) from the Qubit system was included as continuous variables. Coding (e.g. logcfDNA) was confirmed by comparison with restricted cubic spines.

Correlations between the covariables RMH score, age and logcfDNA were assessed visually and the validity of the proportional hazards assumption on performance status and RMH score was investigated by visual assessment of log minus–log survival curves. Statistical analysis was performed in R using the cph-function of the RMS package.^19^ In all cases a *p*-value of 0.05 or less was considered significant. The final model is published as an interactive version using the *Shiny* package in R.^20^ We tested a possible association between cfDNA (quartiles)/RMH score and the risk of not being offered treatment using logistic regression by glm package in R treating both cfDNA quartiles (25th percentile: 7.4 ng/ml; 50th percentile: 17.1 ng/ml; 75th percentile: 39.55 ng/ml) and RMH score as a numerical covariate.

## Results

### Patient characteristics

**Fig. 1:**
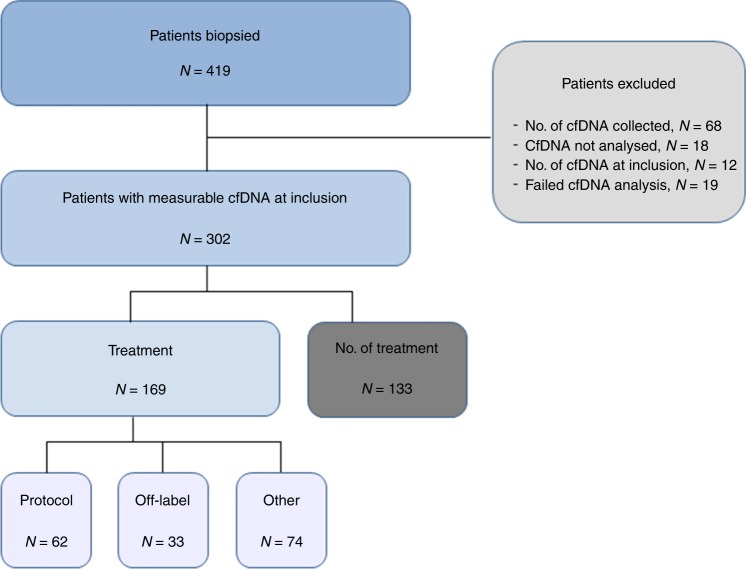
Patient flowchart. A total of 419 patients were included within the study time (3 October 2014 to 22 February 2017) including 302 patients with available plasma cfDNA samples collected at the time of tissue biopsy (baseline). A total of 169 patients were allocated to treatment whereas 133 patients received no further treatment

**Fig. 2:**
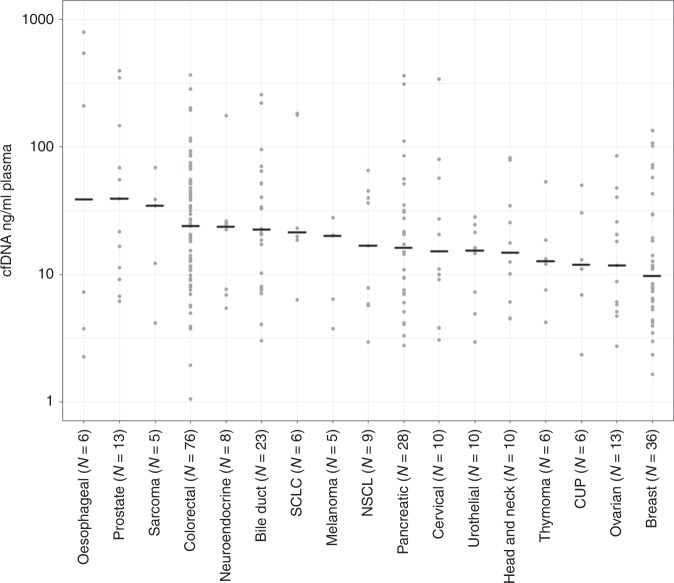
Distribution of cfDNA concentrations (ng/ml plasma) in diverse cancer subtypes. Cancer types including five or more patients are indicated with cfDNA concentrations and the median value for each subtype is indicated by a horizontal line. Each dot represents a patient included in the study

**Fig. 3:**
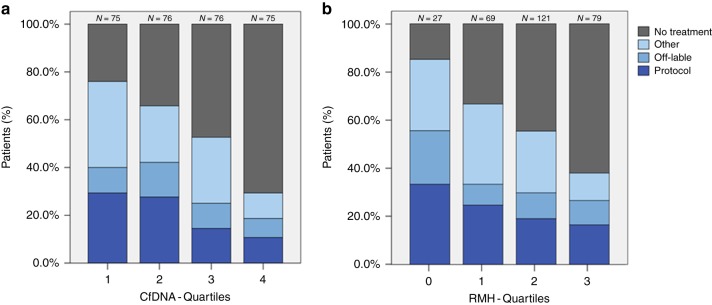
Treatment allocation based on cfDNA concentration (**a**) and RMH score (**b**). The percentage of patients allocated to further treatment or not are indicated based on either cfDNA quantiles (25 percentile: 7.4 ng/ml; 50 percentiles: 17.1 ng/ml; 75 percentiles: 39.55 ng/ml) or RMH score (0–3). Treatment allocation included treatments in a non-phase 1 setting (“Other”), off-label treatment with approved therapy in other diagnosis (“Off-label”) and patients in a phase 1 protocol treatment (“Protocol”). *N* indicates the number of patients in each group. Note that patients are equally distributed in cfDNA quantiles (**a**) in contrast to RMH score (**b**)

A total of 419 patients were included in the CoPPO study within the study period. Measurable plasma cfDNA concentration at inclusion was obtained from 302 patients and 169 patients were allocated to treatment and 133 received no further treatment (Fig. 1). Baseline patient characteristics are summarised in Table 1. There was an equal distribution of gender and age. Patients had received a median of three prior treatment regimens and the majority of patients (63%) presented with more than two metastatic sites. Baseline LDH was elevated in 196 patients (65%) and 168 patients (56%) presented with serum-albumin below normal level. In total, one third of the patients (32%) were classified as having a good prognostic score (RMH score 0–1) with most patients having a performance score of 1 (66%) according to ECOG guidelines. Median OS was 24 weeks (95% CI 21.0–27.0) and median follow-up time of censored cases (*n* = 35) was 84 weeks (CI 73.6–94.4). The most common tumour types were colorectal cancer (25%), followed by breast (12%), pancreatic (9%) and bile duct cancers (8%) (Table S1). Plasma concentration of cfDNA according to cancer subtype is reported in Fig. 2 with an overall median of 17.1 ng/ml (1.1—795.0) (Table S1). Both RMH score and cfDNA quartile was highly associated with the probability of not being offered treatment (Fig. 3, *P* < 0.00001 for both RMH score and cfDNA).

**Table 1:** Patient characteristics

	Total
	(*n* = 302)
*Gender*	
Female	145 (48%)
Male	157 (52%)
*Age*	
Median, range	60 (26–86)
*Metastatic sites*	
**≤**2	111 (37%)
**>**2	190 (63%)
Missing	1 (0%)
*Albumin*	
≥35 g/L	129 (43%)
<35 g/L	168 (56%)
Missing	5 (2%)
*LDH*	
>ULN	196 (65%)
≤ULN	104 (34%)
Missing	2 (1%)
*RMH score*	
0–1	97 (32%)
2–3	200 (66%)
Missing	5 (2%)
*Number of prior treatment regimens*	
Median, range	3 (1–11)
*Performance status (ECOG)*	
0	99 (33%)
1	200 (66%)
2	3* (1%)

*Patients declining in performance status from inclusion to the time of biopsy

### Prognostic value of cfDNA on survival

#### Univariate survival analysis

**Fig. 4:**
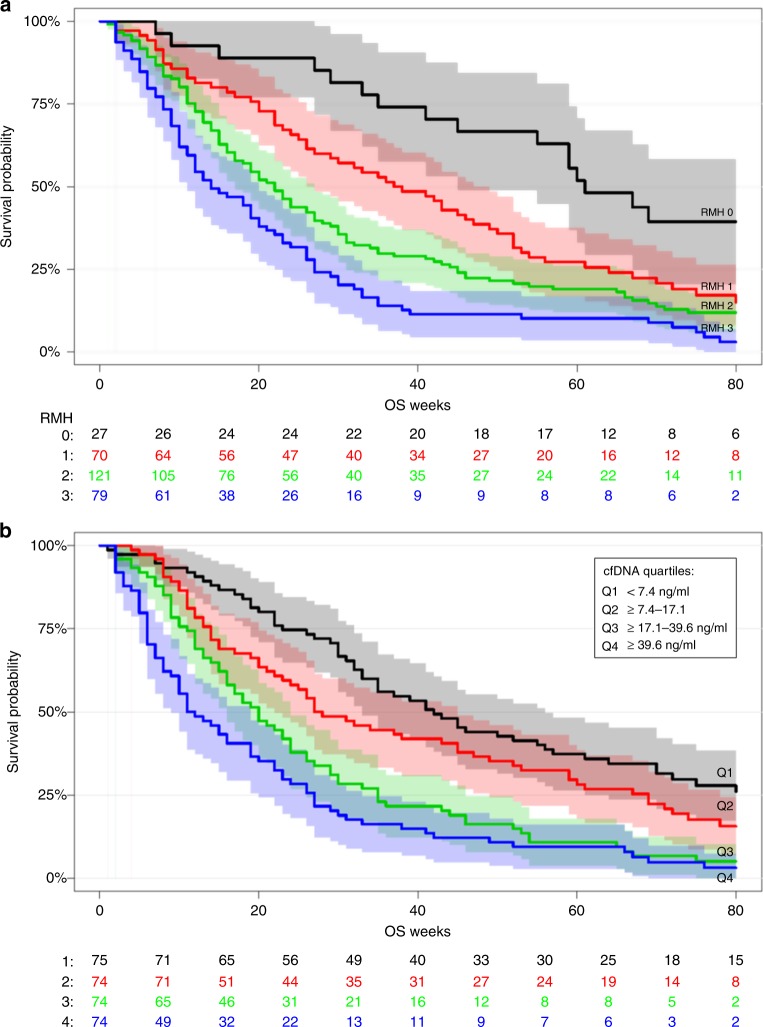
Survival analysis according to RMH score (**a**) and cfDNA level quantiles (**b**). A total of 297 patients were included in the survival analysis. The five patients excluded from the analysis where patients without RMH score (Table 1, patients marked with *). Cell-free DNA levels were split in quartiles

We confirmed a clear association between the RMH score and OS (log-rank *P* < 0.0001) (Fig. 4a). Univariate survival for the four quartiles of cfDNA showed a significant association between cfDNA levels and OS (log-rank *P* < 0.0001) (Fig. 4b). No correlations were observed between RMH score, performance status and the age of the patients. Furthermore, there was no correlation between RMH score and cfDNA (Fig. S1).

#### Multivariate survival analysis

In multivariate analysis including age, RMH, performance status and logcfDNA, low plasma concentration of cfDNA was associated with longer OS with a hazard ratio (HR) of 1.67 (95% CI: 1.39–2.01; *P* < 0.0001). No association between age and survival was observed (Table 2).

**Table 2:** Cox proportional hazards model

	Hazard ratio (95% CI)	*P*-value
Age	0.97 (0.82–1.16)	0.77
cfDNA [log]	1.67 (1.39–2.01)	<0.0001
RMHI 1 vs. 0	2.08 (1.22–3.57)	0.008
RMHI 2 vs. 0	2.29 (1.38–3.80)	0.001
RMHI 3 vs. 0	3.57 (2.12–6.03)	<0.0001
PS 1 vs. 0	2.35 (1.77–3.13)	<0.0001

The OS curves variate according to altering concentrations of cfDNA although RMH score remains the same. An interactive version of the prognostic model is published on http://bit.ly/phase1survival and presented in Fig. S4 by a typical patient from the cohort aged 60 years, RMH score = 2, performance status = 1 and cfDNA levels of 20 and 100 ng/ml plasma.

## Discussion

Clinicians treating patients with exhausted treatment options are facing the dilemma of choosing when to refer the patient to early clinical trials. The RMH score can be used as a prognostic score to guide this decision. In this study, we have shown that by adding the baseline measurement of plasma cfDNA concentration, performance status and age to the RMH score, we were able to build a stronger prediction model assessing OS. This model showed superiority compared to RMH score alone. An improved risk assessment could support the clinical decision when enrolling patients in phase I studies as early discontinuation of patients in phase 1 trials most frequently are due to progression and clinical deteoriation.^21^ The clinical implementation of the proposed model therefore has the potential to improve selection of patients to phase I trials. Furthermore, low plasma concentration of cfDNA at baseline is correlated with a higher chance of receiving further treatment. Similar results were observed with RMH score, suggesting that both markers could be valuable tools in allocating patients to treatment in a phase 1 setting.

Our patient cohort represented a consecutive group of patients included in a molecular profiling project with most patients not receiving treatment within a clinical trial (Fig. 3). It seems likely, that this cohort is representative for a typical cohort of patients referred to phase 1 trials.

However, there are limitations to this study; this is a single centre study and the risk model requires validation in other phase 1 cohorts. Furthermore, we could not exclude whether treatment affected the survival analysis. Low concentrations of cfDNA (< 5 ng/ml) can be measured in healthy individuals mostly emerging from haematopoietic cells^22^ and the concentration of cfDNA can be influenced by other factors including inflammation, infectious disease, exercise etc. These are all confounders of our model and must be considered. For clinical use we would recommend the blood samples to be collected without prior physical activity and notion of an infection. In addition, we only used one method to quantify the cfDNA concentrations. The fluorescence-based Qubit HS assay was selected as this is a simple, fast, cheap and standard method used in most clinical laboratories. Similar assays using intercalating fluorophores have been widely used to detect low levels of cfDNA^18,23^ but other methods are being validated to optimise the accuracy of cfDNA quantification including digital and real-time PCR.^24,25^ Larger studies comparing the different methods are needed to standardise the quantification of cfDNA.

Total cfDNA have previously been shown as a prognostic factor in selected cancer types^13–15^ but, to our knowledge, never used to predict survival in the phase 1 setting across a wide range of cancer types. Additionally, cfDNA has never been added to the RMH score and our data support the addition of cfDNA to improve prediction of survival compared to RMH alone. Olmos et al. have shown that adding the number of circulating tumour cells (CTCs) to the RMH score, improved estimation of OS.^26^ However, this has not been implemented in clinical practice, probably due to the laborious process of isolating CTCs.

In conclusion, we present a model based on a single-centre study, that offers a valuable supplement to the RMH score providing a further improved estimation of the OS for the individual patient. This study supports the continuing efforts exploring the clinical role of cfDNA in cancer care.

## Supplementary information

Supplementary file document

## Data Availability

Data is available as Supplementary material. An interactive version of the prognostic model is published on http://bit.ly/phase1survival
