# Supplementary material for: Plasma total cell-free DNA is a prognostic biomarker of overall survival in metastatic solid tumour patients
Source: Br J Cancer. 2019 Jun 12;121(2):125–30. doi: 10.1038/s41416-019-0491-9 (PMC6738043; doi:10.1038/s41416-019-0491-9)
Supplement: Supplementary file 1 — Supplementary file document [file 41416_2019_491_MOESM1_ESM.pdf]

Figure S1. Scatter plot illustrating the correlation between RMH score, cfDNA concentration (ng/ml plasma) and age of the patients. All patients included in the study (*N*=302) are represented by a dot color coded according to RMH score as illustrated in the legend.

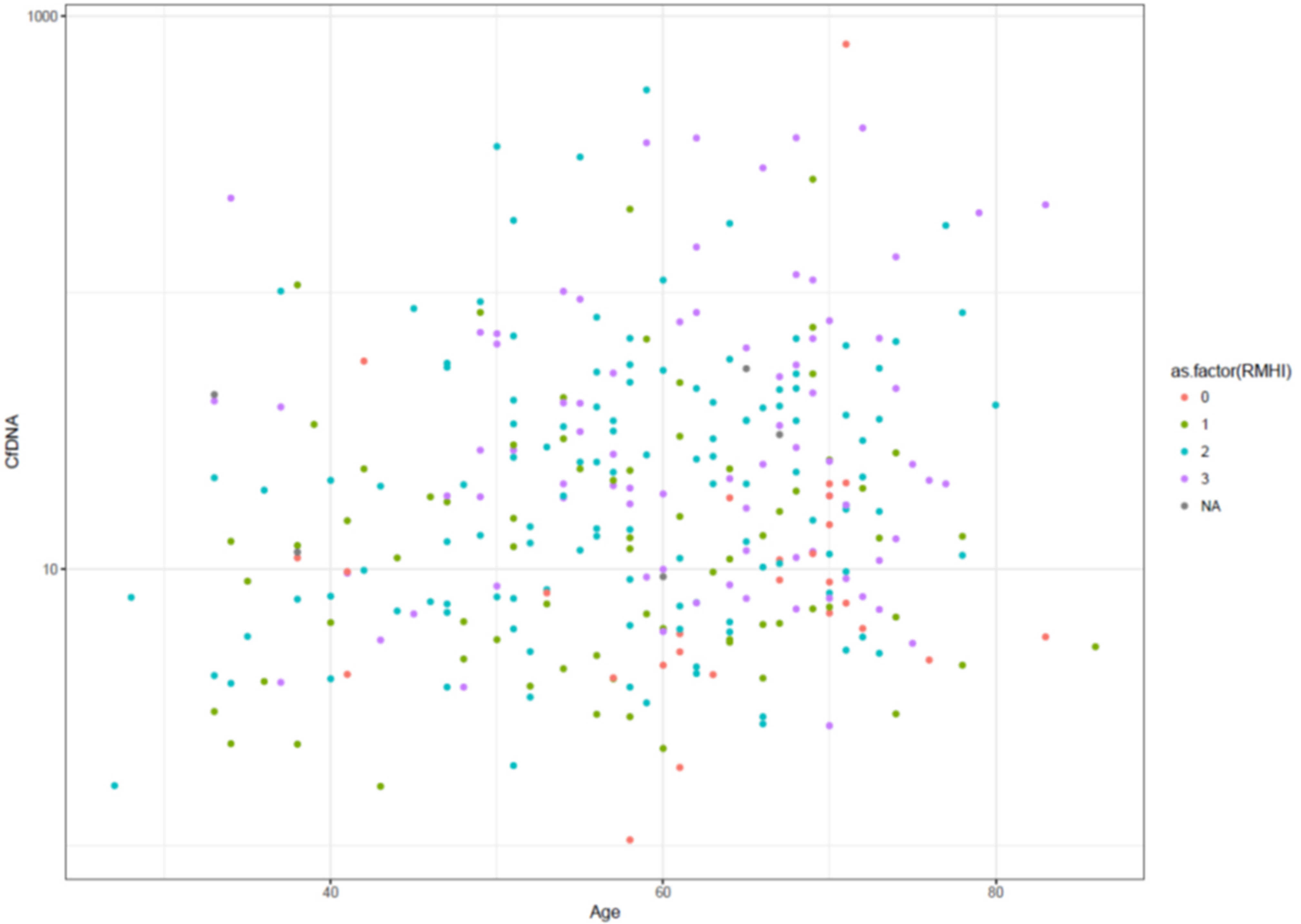
1000
10
CfDNA
40
60
80
Age
as.factor(RMHI)
0
1
2
3
NA

Figure S2. Triplicate measurement of total cfDNA using the Agilent 4200 TapeStation system (D5000). In total 96 samples were quantified, including 49 samples with low cfDNA concentration (0-15 ng/ml plasma (A)) and 47 samples with high concentration (50-1000 ng/ml plasma (B)). For each sample the mean cfDNA concentration (circle) and the standard deviation (lines) are shown. Samples with a concentration >100 ng/ml plasma were diluted (1:10) before measurement on the TapeStation system.

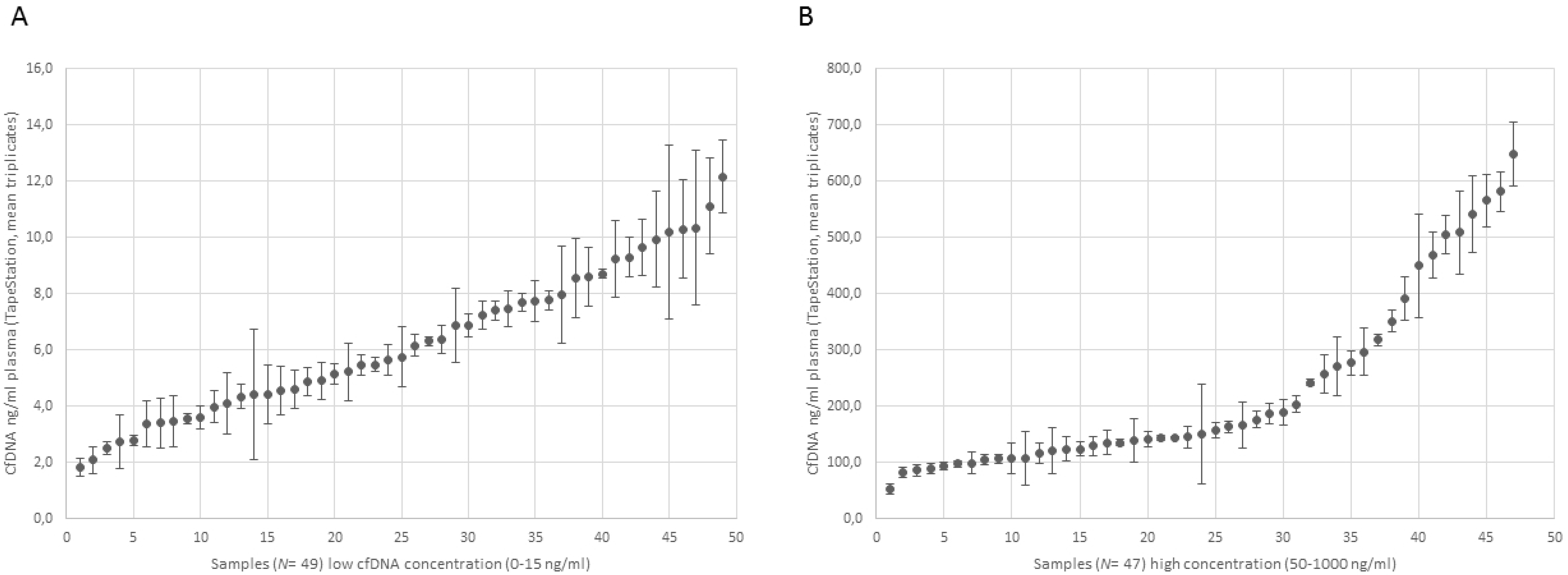
A
B
CfDNA ng/ml plasma (TapeStation, mean triplicates)
Samples (N= 49) low cfDNA concentration (0-15 ng/ml)
Samples (N= 47) high concentration (50-1000 ng/ml)
16,0
14,0
12,0
10,0
8,0
6,0
4,0
2,0
0,0
800,0
700,0
600,0
500,0
400,0
300,0
200,0
100,0
0
5
10
15
20
25
30
35
40
45
50

Figure S3. A) Concordance between Qubit and TapeStation cfDNA quantification (ng/ml plasma). Each circle represents a sample (*N*=96). The total cfDNA concentration from the TapeStation system represents the mean value from the triplicate measurements shown in Fig. S2 divided by two. This is due to the difference in quantification technology. The TapeStation system uses electrophoresis to estimate the concentration of cfDNA whereas Qubit uses intercalating fluorescent dyes binding only double stranded DNA, leading to concentration measures around half the value of the ones from the TapeStation. This was supported by the Bland-Altman diagram (B). The mean of the two methods is plotted as the x-coordinate, and the %-difference in cfDNA concentration ((TapeStation/2-Qubit)/Qubit)*100%) as the y-coordinate. The mean of all %-differences (21.75%) is indicated by a solid black line and the 95% limits of agreement are indicated by two gray lines, at a distance of 1.96 times the standard deviation of the differences.

A

B

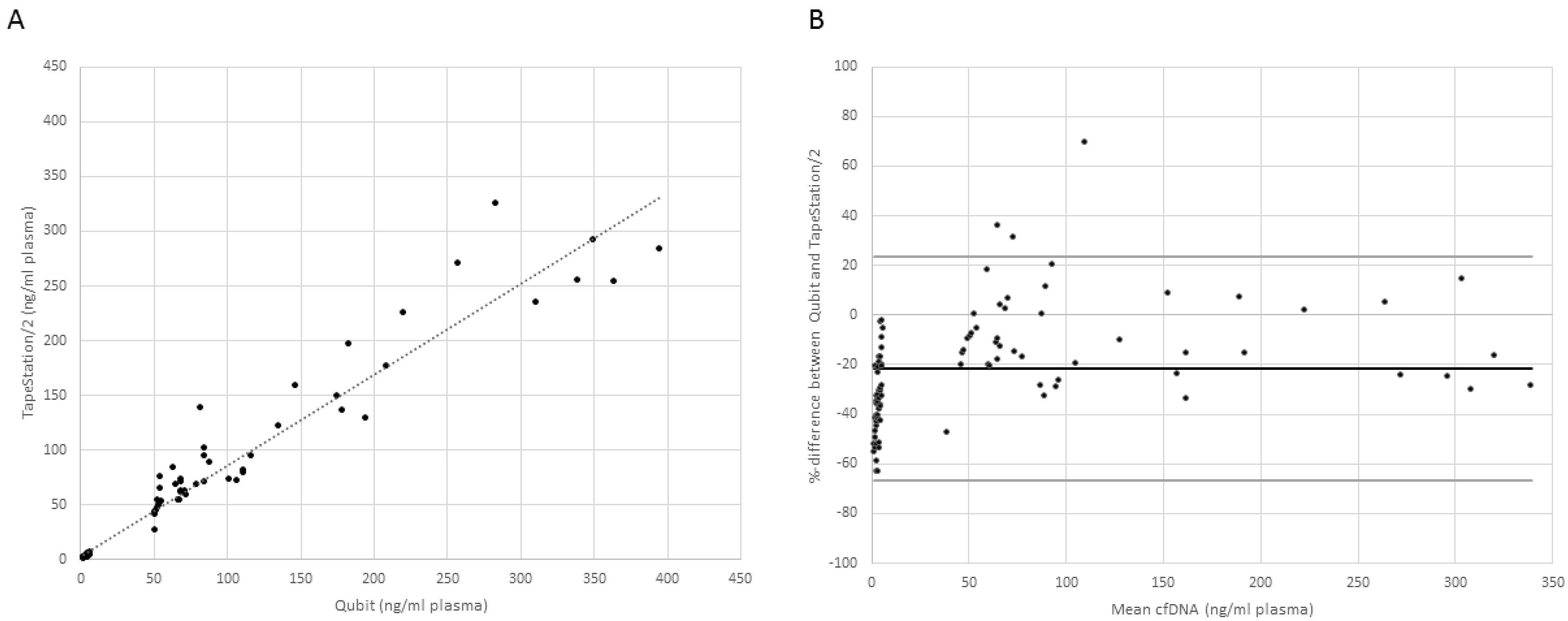
A
TapeStation/2 (ng/ml plasma)
Qubit (ng/ml plasma)
0
50
100
150
200
250
300
350
400
450
B
%-difference between Qubit and TapeStation/2
Mean cfDNA (ng/ml plasma)
100
80
60
40
20
0
-20
-40
-60
-80
-100
0
50
100
150
200
250
300
350

Figure S4. Illustration of the interactive version of the multivariable model. Predicted survival is shown for a 60-year old reference CoPPO patient with RMH score =2, performance status =1 and cfDNA levels of 20 ng/ml (A) and 100 ng/ml plasma (B).

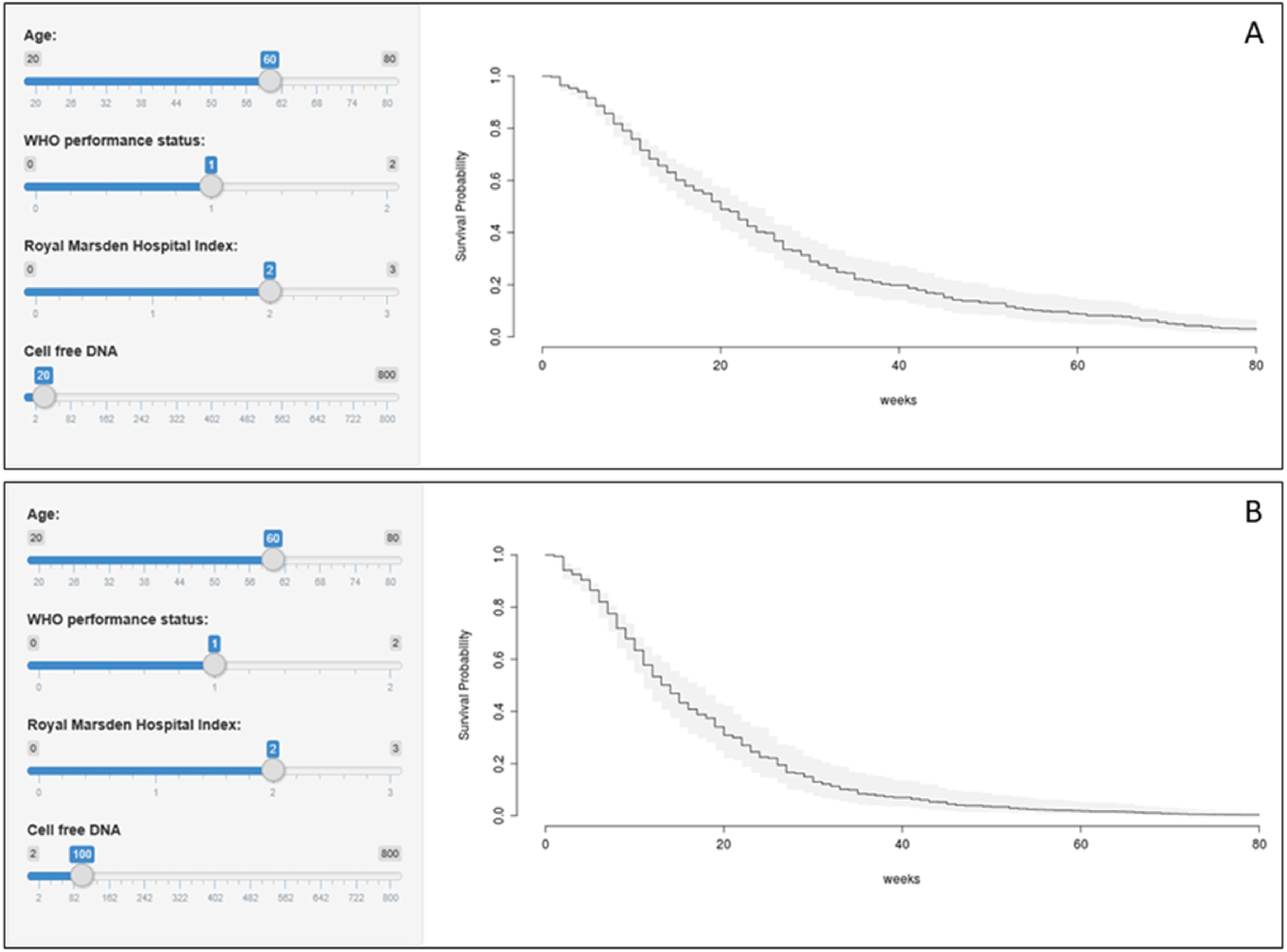
A
Age:
20
60
80
20 26 32 38 44 50 56 62 68 74 80
WHO performance status:
0
1
2
Royal Marsden Hospital Index:
0
2
3
0 1 2 3
Cell free DNA
20
800
2 82 162 242 322 402 482 562 642 722 800
Survival Probability
0.0 0.2 0.4 0.6 0.8 1.0
0 20 40 60 80
weeks
B
Age:
20
60
80
20 26 32 38 44 50 56 62 68 74 80
WHO performance status:
0
1
2
Royal Marsden Hospital Index:
0
2
3
0 1 2 3
Cell free DNA
2
100
800
2 82 162 242 322 402 482 562 642 722 800
Survival Probability
0.0 0.2 0.4 0.6 0.8 1.0
0 20 40 60 80
weeks

Table S1. Cohort overview including cancer subtypes and cfDNA level.

**Cancer subtype**	**Number of patients (*N*)**	**Median cfDNA (ng/ml plasma)**	**cfDNA range (ng/ml plasma)**
Colorectal cancer	76	24.0	1.1 – 364.5
Breast cancer	36	9.7	1.6 – 135.2
Pancreatic cancer	28	16.1	2.8 – 363.8
Bile duct cancer	23	22.5	3.0 – 258.0
Ovarian cancer	13	11.7	2.7 – 84.9
Prostate cancer	13	39.0	6.1 – 395.5
Cervical cancer	10	15.8	3.1 – 339.0
Head and Neck cancer	10	15.0	4.4 – 81.8
Urothelial cancer	10	15.3	2.9 – 28.2
NSCLC	9	16.7	2.9 – 65.4
Neuroendocrine cancer	8	23.5	5.4 – 175.5
SCLC	6	21.4	6.3 – 183.0
Thymoma	6	12.6	4.2 – 53.3
Oesophageal cancer	6	107.8	2.3 – 795.0
CUP	6	11.9	2.3 – 49.8
Melanoma	5	20.1	3.8 – 27.7
Sarcoma	5	34.5	4.1 – 68.4
Gastric cancer	4	17.9	4.7 – 63.3
Hepatocellular carcinoma	4	20.1	13.3 – 53.4
Adrenal gland cancer	4	17.1	9.3 – 101.6
Mesothelioma	4	13.8	5.5 – 10.5
Renal cell carcinoma	3	7.4	5.9 – 10.5
Adenoid cystic carcinoma	2		4.5 and 21.0

Endometrial cancer	2		7.9 and 11.9
Anogenital cancer	2		6.5 and 9.7
Myoepithelial carcinoma	2		4.1 and 5.0
CNS tumor	1		16.2
Germ cell tumor	1		1.7
Others	3	4.0	1.9 – 33.5
- Granulosecelletumor	2		1.9 and 4.2
- Malign myxoid tumor	1		33.5
